# *Streptomyces* Dominate the Soil Under *Betula* Trees That Have Naturally Colonized a Red Gypsum Landfill

**DOI:** 10.3389/fmicb.2018.01772

**Published:** 2018-08-03

**Authors:** Cyril Zappelini, Vanessa Alvarez-Lopez, Nicolas Capelli, Christophe Guyeux, Michel Chalot

**Affiliations:** ^1^Laboratoire Chrono-Environnement, UMR CNRS 6249, Université Bourgogne Franche-Comté, Montbéliard, France; ^2^Département d’Informatique des Systèmes Complexes, Institut FEMTO-ST (UMR 6174 CNRS), Université Bourgogne Franche-Comté, Besançon, France; ^3^Faculté des Sciences et Technologies, Université de Lorraine, Nancy, France

**Keywords:** *Actinobacteria*, red gypsum landfill, birch, plant growth promoting traits, biological restoration

## Abstract

The successful restoration of well-engineered tailings storage facilities is needed to avoid mine tailings problems. This study characterized the bacterial communities from vegetated and non-vegetated soils from a red gypsum landfill resulting from the industrial extraction of titanium. A set of 275 bacteria was isolated from vegetated soil and non-vegetated soil areas and taxonomically characterized using BOX-PCR. The study also evaluated the ability of a subset of 88 isolated bacteria on their ability to produce plant growth promoting (PGP) traits [indoleacetic acid (IAA) production, phosphate solubilization, and siderophore production] and their tolerance to potentially toxic elements (PTEs). Twenty strains were chosen for further analysis to produce inoculum for birch-challenging experiments. Principal component analysis (PCA) showed that the set of pedological parameters (pH, granulometry, carbon, organic matter, and Mg content) alone explained approximately 40% of the differences between the two soils. The highest density of total culturable bacteria was found in the vegetated soil, and it was much higher than that in the non-vegetated soil. The *Actinobacteria* phyla dominated the culturable soil community (70% in vegetated soil and 95% in non-vegetated soil), while the phyla *Firmicutes* (including the genus *Bacillus*) and *Bacteroides* (including the genera *Pedobacter* and *Olivibacter*) were found only in the vegetated soil fraction. Additional genera (*Rhizobium, Variovorax*, and *Ensifer)* were found solely in the vegetated soil. The vegetated soil bacteria harbored the most beneficial PGP bacteria with 12% of the isolates showing three or more PGP traits. The strains with higher metal tolerances in our study were *Phyllobacterium* sp. WR140 (RO1.15), *Phyllobacterium* sp. WR140 (R01.34), and *Streptomyces* sp. (R04.15), all isolated from the vegetated soil. Among the isolates tested in challenging experiments, *Phyllobacterium* (R01.34) and *Streptomyces* sp. (R05.33) have the greatest potential to act as PGP rhizobacteria and therefore to be used in the biological restoration of tailings dumps.

## Introduction

Mining operations produce significant volumes of waste substrates that originated from the physical or chemical treatment of waste rocks from which ores have been extracted (Kumaresan et al., 2017). Such extractive (anthropogenic) activities produce potentially toxic elements (PTEs) that may contaminate the environment and induce human health problems. Artificial substrates generated by these extractive activities have not been subjected to weathering (Cross et al., 2017) and represent substantial volumes of wastes that are vulnerable to water and wind erosion, and thus potentially transported to long distances (Honeker et al., 2017). They are frequently characterized by a poor physico-hydrological structure, resulting in an unstable geochemical nature, and the presence of potentially toxic chemicals (Wang et al., 2017). Factually, management of tailings plans have often been concentrated on their confinement and containment and little attention has been paid to the long-term alteration of the tailings materials, including changes of their biological, chemical or physical, properties (Santini and Banning, 2016). In addition, they contain abundant by-products, which could be potentially used as amendment in land farming, although they obviously encompass substantial abiotic constraints for the survival of plant and microorganisms.

Revegetation is often encouraged on these tailings since it can efficiently control the erosion of tailings particles by wind and water and may advance the landscape of these waste areas (Mendez and Maier, 2008). The successful restoration of tailings storage amenities may be indeed the best technique to limit mine tailings tragedies (Cross et al., 2017). Salisbury et al. (2017) demonstrated the effectiveness of a vegetative cover to retain some PTE in the upper soil horizons for some decades, and thus playing a considerable function in reclaiming contaminated land. Plant may indeed tolerate PTE contamination through various mechanisms (exclusion, hyperaccumulation traits, and microbe functions) that allow growth and reproduction in such severe environments. Nonetheless, the *in situ* remediation of tailings is likely to necessitate the addition of amendments that may accelerate substrate weathering (Santini and Banning, 2016). The addition of a topsoil to the rooting soil area represents an efficient method to alleviate the abiotic constraints existing in original tailings and is likely to hasten the reappearance of microbial functions (Huang et al., 2012). Choosing plant species that are endemic to the tailings areas is recognized as a suitable choice for a successful revegetation (Wang et al., 2008; Jana et al., 2012; Wanat et al., 2014). Among plant species that readily colonize tailings, *Betula* species have a recognized ability to quickly colonize bare areas and are characterized by their poor affinity for any specific soil category and their capacity to grow in nutrient poor substrates (Atkinson, 1992; Jana et al., 2012).

There is abundant literature on the characterization of microbial communities from forest or agricultural soils contaminated by PTE or polycyclic aromatic hydrocarbon (PAH) (Tardy et al., 2015; Yergeau et al., 2015; Foulon et al., 2016a,b). Microorganisms occurring in mine tailings have also drew significant interest in the past decade, especially in acid mine drainage dumps (Méndez-García et al., 2015; Bruneel et al., 2017; Gupta et al., 2017; Mesa et al., 2017). However, in other environments with different soil characteristics (bauxite and red gypsum), there are considerably fewer studies. Microorganisms are both relevant indicators of ecological functions and facilitators of the soil metabolic activities that are required for further aboveground-plant reestablishment. However, the mechanisms involved on how microorganisms facilitate restoration of degraded lands such as post-mining lands remain poorly understood. Wubs et al. (2016) reported that the addition of microbial inoculum could foster ecosystem restoration, while emphasizing that the origin of the inoculum was a major factor to promote the establishment of plant communities. Therefore, efforts to characterize endogenous microbial communities from these soils are urgently needed to achieve optimal plant recovery.

Among soil bacteria, *Actinobacteria* constitute a group of microorganisms found in high amounts in soils and play key roles in the recycling of natural compounds or xenobiotic such as pesticides and PTE, due to their metabolic capacity (Kieser et al., 2000). Currently, *Actinobacteria* are considered among the most prosperous colonizers in most extreme environments, in contrast to being conventionally considered as endogenous soil and freshwater microorganisms (Álvarez et al., 2017). *Actinobacteria* can directly promote plant growth by supplying the plant with bacterial-synthesized compounds or by facilitating soil nutrient uptake by the plant (Barka et al., 2016). Actinobacteria may also prevent infection by deleterious microorganisms, which is achieved through biocontrol or antagonism toward soil plant pathogens. Despite these recognized traits, the plant growth promoting (PGP) rhizospheric potential of *Streptomyces* has been poorly studied, although there are widely recognized as efficient root colonizers and able to cope with unfavorable growth conditions by forming spores. *Actinomycetes* strains were isolated from birch rhizospheric soils, birch being one of the few native tree able to succeed on a coal mine dump (Ostash et al., 2013).

The present work focused on plant root microbe interactions occurring in a titanium tailings dump that has been naturally recolonized by birch trees, to increase our understanding on how these interactions may be favorable to plant redeployment on such stressful environment. The primary objectives of the present study were: (i) to isolate indigenous bacteria from birch (*Betula* spp.) based on physiological and morphological traits as well as using 16S rRNA gene sequencing, (ii) to test PGP functional traits from isolated bacteria, such as indoleacetic acid (IAA) production, siderophore production, phosphate solubilization and metal tolerance, and (iii) to study the PGP potential of bacterial isolates under controlled conditions.

## Materials and Methods

### Study Site Location

The study site belongs to an 80 ha titanium industry effluent treatment unit located at Thann in northeastern France in the southern part of the Alsace plain (47°47′47.7″N 7°08′18.5″E). The study was carried out in a tailing dump consisting of an embankment, where byproducts produced during the neutralization of titanium dioxide extraction effluents have been stored since the 1930s. The dump surface studied here has not been used since the early 2000s, which has allowed natural revegetation with flora that is not very abundant and is distributed in a heterogeneous way. We may thus observe heavily vegetated areas and, in contrast, areas completely bare of vegetation. The flora at this dumpsite is almost exclusively dominated by the woody species *Betula* sp.

### Sampling

**FIGURE 1:**
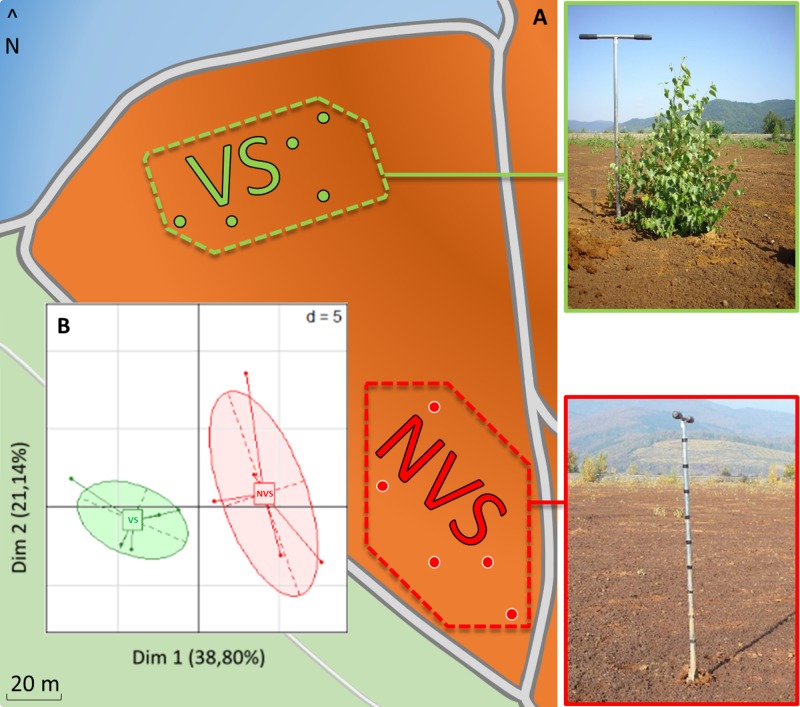
**(A)** Diagram of the sampling site with soils taken in a vegetated area under birch trees (VS, top-left photo, green dotted line) or soil taken in a non-vegetated area (NVS, bottom-left photo). **(B)** PCA carried out with soil data and soil element concentrations discriminating between the two sampling areas, *n* = 5.

The samples were collected on October 27, 2015. They consisted of samples from two areas, a vegetated and a non-vegetated areas (**Figure 1**). Five birches distributed over the vegetated area were harvested, and the soil fraction adhering to the root system was collected (vegetated soil or VS). For the non-vegetated area, five samples (non-vegetated soil or NVS) were also sampled using an auger at a depth approximately close to that of the root system for the vegetated area. The whole samples were packed on site in plastic bags and transported to the laboratory at a temperature approaching 4°C.

### Pedological Characterization

The soils were dried at 40°C and then ground by hand at 2 mm. The soil analyses were carried out by a service provider in accordance with the following French standards for grinding (NF ISO 11464), residual humidity (NF ISO 11465), granulometry (5 fractions – NFX 31-107), pH water + KCl (NF ISO 10390), total organic carbon and organic matter (NF ISO 14235), total nitrogen (NF ISO 13878), CEC Metson (NFX 31-130), bore soluble boiling water (NFX 31-122), oligoelements, K_2_O, MgO, CaO, Na_2_O (French Norm X 31-108), and total available phosphorus (Joret Hebert method French Norm X 31-161).

In addition, pseudo-total concentrations in the soils were measured using inductively coupled plasma atomic emission spectrometry (ICP-AES, Thermo Fischer Scientific, Inc., Pittsburgh, PA, United States) analysis after the acid digestion of 500 mg of a sample in a microwave digestion system (Mars Xpress, CEM, Saclay, France), using a mix of 2 mL of 67% nitric acid, 6 mL of 34% hydrochloric acid, and 2 mL of 48% hydrofluoric acid. To assess the analytical quality, a standard reference material (loamy clay soil) was used. To determine the extractable fractions of PTE, 5 g of 2 mm sieved soil was dried at 60°C for 48 h (or air-dried) and incubated with 50 mL of 10 mM CaCl_2_ under agitation (40 rpm) for 2 h at room temperature. The mixture was first filtered with ash-free filters, passed hrough a 0.45 μm mesh, and acidified at 2% (v/v) with HNO_3_ prior to ICP-AES analysis.

### Microbial Characterization

The vegetated and non-vegetated soil fractions were homogenized in 45 ml of 10 mM MgSO_4_ and stirred at 100 rpm for 15 min at room temperature. One milliliter was used to perform serial dilutions in 10-fold series, and 100 μL was plated onto a 284-agar medium (Becerra-Castro et al., 2011b) in duplicate dilutions and kept for 7 days at 25°C. The 284 medium contains (per liter): 6.06 g Tris–HCl, 4.68 g NaCl, 1.49 g KCl, 1.07 g NH_4_Cl, 0.43 g Na_2_SO_4_, 0.2 g MgCl_2_.6H_2_O, 0.03 g CaCl_2_.2H_2_O, 0.04 g Na2HPO4.2H2O, and 10 mL Fe(III)NH4 citrate solution (containing 48 mg/100 mL) plus micronutrients (1.5 mg FeSO_4_.7H_2_O, 0.3 mg H_3_BO_4_, 0.19 mg CoCl_2_.H_2_O, 0.1 mg MnCl_2_.4H_2_O, 0.08 mg ZnSO_4_.7H_2_O, 0.02 mg CuSO_4_.5H_2_O, and 0.036 mg Na_2_MoO_4_.2H_2_O) adjusted to a pH of 7. The medium was supplemented with a mixture of different carbon sources: lactate (0.7 g/L), glucose (0.5 g/L), gluconate (0.7 g/L), fructose (0.5 g/L), and succinate (0.8 g/L). Culturable bacterial densities were calculated and expressed as CFU per gram dry soil. Single morphotypes were isolated by plating them twice onto 284 medium-agar plates. The isolates were further stored in cryotubes in a brain heart infusion broth (Roth, D) with 15% glycerol glucosate at −80°C.

### Genotypic Characterization

#### DNA Extraction and BOX-PCR

For DNA preparation, the isolates were grown in the 284 liquid medium at 25°C for 7 days at 250 rpm (Gallenkamp Orbital Incubator). After centrifugation, DNA was extracted from the pellets using an EZNA bacterial DNA isolation kit (Omega Bio-Tek, Inc., Norcross, GA, United States) according to the manufacturer’s instructions. The BOX-PCR fingerprinting method was used to group genotypic profiles at a similarity level of 90% as previously described (Becerra-Castro et al., 2011a). BOX reactions were performed in a reaction volume of 25 μL containing 12.5 μL of Ready Mix PCR Master Mix (Thermo Fisher Scientific, Carlsbad, CA, United States), 2 μM BOX A1R primer (5′- CTACGGCAAGGCGACGCTGACG-3′, Eurofins Genomics, Paris, France), and 5 μL of bacterial DNA. DNA amplification was carried out in a thermocycler (Mastercycler gradient, Eppendorf, Hamburg, Germany) under the following conditions: 1 cycle of 5 min at 95°C, 40 cycles of 25 s at 95°C, 35 s at 55°C, and 1.05 min at 72°C with an additional 5 min cycle at 72°C. The amplicons obtained were separated by electrophoresis on a 1.8% agarose gel at 45 V for 3 h. The gel images were analyzed with the software Gel.J (Heras et al., 2015) using the Pearson correlation coefficient and a UPGMA clustering algorithm. VS and NVS bacteria were treated separately.

#### 16S Taxonomic Assignment

PCR was performed on one representative of each BOX group using the following conditions: a volume of 50 μL containing 25 μL AccuStartTM II PCR ToughMix^®^ (2×) (Quantas), 5 μM 27f (*Escherichia coli* positions 8–27, 5′-AGAGTTTGAT CCTGGCTCAG-3′) and 1492r (*E. coli* positions 1,492–1,510, 5′-ACGGTTACC TTGTTACGACTT-3′), which were used to amplify nearly full-length 16S rRNA genes (Mark Ibekwe et al., 2007), and 5 μL of cell lysate. The thermocycling conditions were as follows: 1 cycle of 94°C for 3 min, 40 cycles of 25 s at 94°C, 25 s at 49.4°C, and 1.30 min at 72°C. Alignments were performed using the SILVA website^1^.

### Functional Traits

#### Characterization of PGP Traits

Isolates were screened for their ability to solubilize inorganic phosphate and the production of siderophores, organic acids, and IAA. All of these analyses were conducted for one representative of each BOX group. The ability to solubilize inorganic phosphate was assessed in a modified NBRIP agar medium (1.8%) supplied with 5 g/L of hydroxyapatite and incubated at 28°C for 5 days [10.0 g glucose, 5.0 g MgCl_2_.6H_2_O, 0.25 g MgSO_4_.7H_2_O, 0.2 g KCl, 0.1 g (NH_4_)_2_SO_4_, and 0.1 g yeast extract in 1 L deionized water adjusted to a pH of 7.0 modified from Nautiyal, 1999]. A clear halo around the bacterial colony indicated the solubilization of mineral phosphate. Siderophore production was detected in a modified 284 liquid medium (without Fe) using the Chrome Azurol S (CAS) method described by Schwyn and Neilands (1987). All glassware used in this assay was previously cleaned with 30% HNO_3_ followed by washing in distilled water (Cox, 1994).

The ability to produce organic acids was tested on an agar medium containing 0.002% bromocresol purple (per liter medium): 10.0 g glucose, 1.0 g tryptone, 0.5 g yeast extract, 0.5 g NaCl, and 0.03 g CaCl_2_.2H_2_O. Colonies forming a yellow halo after 1 day of growth at 28°C indicated a pH change in the medium, and they were considered acid producers. IAA production was evaluated in liquid medium [5.0 g glucose, 1.0 g (NH_4_)_2_SO_4_, 2.0 g K_2_HPO_4_, 0.5 g CaCO_3_, 0.5 g MgSO_4_.7H_2_O, 0.1 g NaCl, and 0.1 g yeast extract adjusted to a pH of 7 modified from Sheng et al. (2008); supplemented with 0.5 mg/mL tryptophan]. After 5 days incubation at 28°C, the cultures were centrifuged, and the supernatant was incubated with the Salkowski reagent for 25 min. The production of IAA was identified by the presence of red coloring, and isolates were considered IAA producers when the concentration of IAA determined was more than 4 mg/L culture.

#### Metal Tolerance

Metal tolerance was tested for Cr, Mn, and Zn using 284 agar medium (see above) supplemented with increasing concentrations of Cr [0.1, 0.25, 0.5, 1.0, 2.5, and 5.0 mM; added as Cr(NO_3_)_3_.9H_2_O], Zn [1.0, 2.5, 5.0, 10.0, and 25.0 mM added as Zn(SO_4_)_2_.7H_2_O] and Mn [5.0, 10.0, and 25.0 mM added as MnSO_4_.H_2_O] and incubated at 28°C for 7 days. The maximal tolerable concentration (MTC) of each metal was recorded for one selected isolate of each BOX-group.

### Plant Inoculation Experimental Setup and Post-harvest Analysis

Birch seeds were germinated in a commercial potting mixture. Three-month-old birch seedlings were transplanted into pots containing 200 g of soil collected from the study site. After 1 week of plant adaptation, bacterial inoculation was carried out. Fresh cultures of bacterial strains were grown in an 869 liquid medium (Mergeay et al., 1985) for 24 h, harvested by centrifugation (6,000 rpm, 15 min) and re-suspended in 10 mM MgSO_4_ to a dry mass weight of 0.5 mg/L. Each pot was inoculated with 10 mL of bacterial suspension. The same amount of sterile 10 mM MgSO_4_ was added to the non-inoculated pots. Six replicates of each plant species were prepared for each inoculation strain. Plants were watered regularly to maintain soil moisture and incubated in a growth chamber in the following climatic conditions: daylight for 16 h (250–300 μmol m^−2^ s^−1^), day temperature of 22°C, night temperature of 18°C, and day and night humidities of 30%.

After a 3 months growth period, the plants were harvested and the shoot and root dry weight (DW) yields were determined. The plant material was washed in deionized water, oven-dried at 45°C, weighed and ground. The oven-dried plant material was digested in a 2:1 HNO_3_:HCl mixture, and the concentrations of P, K, Ca, Mg, Fe, Cd, Pb, and Zn were measured by ICP-AES.

### Statistical Analyses

All statistical analyses were performed using R software v. 3.0.2 (R Core Team, 2013). Normality was tested with using Shapiro-Wilk (all data sets), and homoscedasticity was tested with Bartlett’s (abiotic dataset) and Levene (biomass dataset, PGP, and metal tolerance traits) tests using R. Data that were normally distributed were analyzed using a parametric test (Student’s *t*-test) in R. Data that were not normally distributed were analyzed using a non-parametric Mann–Whitney–Wilcoxon (soil data) or a Kruskal–Wallis (inoculation) test using R. The principal component analysis (PCA) was performed using the R ade4 package. Data expressed as % (PGP and metal tolerance traits) were analyzed using a chi-squared test in R.

## Results and Discussion

### Pedological Characterization of the Two Areas

The sampling zone where the vegetation was found (*Betula pendula*) is separated by approximately 60 m from the non-vegetated area (**Figure 1A**). Investigations carried out at the physico-chemical level show that the VS fraction differed significantly from the NVS fraction in several pedological parameters (**Table 1**) and elements (**Table 2**). Physico-chemical analysis revealed that the VS contained significantly less silt and more sand (**Table 1**) and was slightly more acidic than the NVS. It also contained more C and OM. Significant differences between the NVS and VS samples were found for the following parameters: Ti (+25.40% in VS), Mn (+60.04% in NVS), K (+27.66% in NVS), Sb (+28.88% in NVS), As (+32.43% in NVS), and B (+33.31% in NVS). In the CaCl_2_ extractable fraction, only B, Cr, Fe, K, Mg, Mn, Na, P, S, Si, Sr, Ti, and Zn were detected in significant amounts in this fraction (>0.01% from the total) (**Table 2**). However, for Fe, Mn, and Ti, the extractable fraction accounted for less than 0.01%. Conversely, for Cr, K, Mg, Si, S, and Sr, the extractable fraction accounted for approximately 2–10%. Only the total concentrations of As, B, Mg, Sr, Sb, and Ti differed significantly between the VS and NVS samples, while only the CaCl_2_ extractable fraction of Mg differed between the two soils.

**Table 1:** Physico-chemical parameters of vegetated soils (VS) and non-vegetated soils (NVS).

	Non-vegetated soil	Vegetated soil	*P*-value	Test value
Clay (bbb)	100.40 ± 6.73	93.60 ± 5.37	0.117	*t* = −1.766
Thin silt (bbb)	228.60 ± 38.72	152.20 ± 23.97	0.007^∗∗^	*t* = −3.751
Large silt (bbb)	220.20 ± 17.91	138.80 ± 13.63	5.95E-05^∗∗∗^	*t* = −8.088
Thin sand (bbb)	169.20 ± 45.65	157.20 ± 29.99	0.638	*t* = −0.491
Coarse sand (bbb)	281.80 ± 57.22	458.40 ± 26.90	0.0009^∗∗∗^	*t* = 6.245
pH	8.20 ± 0.35	7.84 ± 0.05	0.007^∗∗^	*W* = 25
pH KCl	8.08 ± 0.36	7.84 ± 0.05	0.009^∗∗^	*W* = 25
CaCO_3_ (g/kg)	280.40 ± 115.79	301.60 ± 51.23	0.722	*t* = 0.374
C. org (g/kg)	3.78 ± 0.87	6.88 ± 1.70	0.011^∗^	*t* = 3.623
OM (g/kg)	6.54 ± 1.48	11.90 ± 2.95	0.011^∗^	*t* = 3.626
N tot (g/kg)	<ddl	<ddl		
C/N	NC	NC		
CEC (meq/kg)	26.80 ± 6.06	45.20 ± 11.21	0.017^∗^	*t* = 3.228
K_2_Oex (g/kg)	0.00 ± 0.00	0.01 ± 0.01	0.07201	*W* = 5
MgOex (g/kg)	0.76 ± 0.20	0.30 ± 0.16	0.004^∗∗^	*t* = −3.987
CaOex (g/kg)	76.84 ± 0.44	77.02 ± 0.36	0.500	*t* = 0.706
Na_2_Oex (g/kg)	0.02 ± 0.00	0.02 ± 0.00	0.204	*t* = −1.407

*Mean values and standard deviations are provided (n = 5). Normally distributed data were analyzed using the parametric Student’s t-test (t-values). Data not normally distributed were analyzed using the non-parametric Mann–Whitney–Wilcoxon (W-values). Significant differences are indicated (^∗^P < 0.05; ^∗∗^P < 0.01; and ^∗∗∗^P < 0.001).*

**Table 2:** Total and CaCl_2_ extractable element concentrations in vegetated soils (VS) and non-vegetated soils (NVS).

	NVS		VS		Statistics			
	Total (ppm)	CaCl_2_ extractable (ppm)	Total (ppm)	CaCl_2_ extractable (ppm)	Total		CaCl_2_ extractable	
					*P*-value	Test value	*P*-value	Test value
Al	2,708 ± 158		3,010 ± 469		0.309	*W* = 7		
As	6.11 ± 1.24		4.13 ± 0.89		0.022**^∗^**	*t* = −2.90		
B	5.13 ± 2.68	0.61 ± 0.21	3.42 ± 1.27	0.40 ± 0.12	0.015**^∗^**	*t* = −3.10	0.101	*t* = −1.91
Ca	194,267 ± 19462		186,187 ± 8,115		0.341	*t* = −1.04		
Cd	0.21 ± 0.21		0.15 ± 0.21		0.824	*W* = 14		
Co	16.24 ± 2.53		21.54 ± 4.33		0.077	*t* = 2.11		
Cr	147.48 ± 43.06	8.26 ± 4.73	186.10 ± 27.67	3.79 ± 0.49	0.156	*t* = 1.60	0.151	*W* = 20
Cu	38.50 ± 2.78		45.78 ± 7.03		0.118	*t* = 1.87		
Fe	41,559 ± 4,542	0.99 ± 0.24	52,198 ± 9,242	1.13 ± 0.37	0.088	*t* = 2.04	0.526	*t* = 0.667
K	156.23 ± 17.91	6.97 ± 1.53	113.02 ± 31.47	10.39 ± 4.03	0.041**^∗^**	*t* = −2.58	0.136	*t* = 1,774
Mg	4,063 ± 1,336	330.87 ± 67.62	1,623 ± 372.30	141.35 ± 71.13	0.001**^∗∗^**	*t* = −4.88	0.002**^∗∗^**	*t* = −4.318
Mn	3,167 ± 538.06	0.19 ± 0.05	4,228 ± 1,023	0.16 ± 0.05	0.112	*t* = 1.863	0.409	*t* = −0.872
Na	76.99 ± 24.88	8.70 ± 1.58	98.54 ± 26.59	7.99 ± 1.75	0.059	*t* = 2.397	0.521	*t* = −0.671
Ni	22.15 ± 16.81		24.61 ± 18.15		0.471	*t* = 0.770		
P	45.57 ± 22.63	0.14 ± 0.01	47.18 ± 24.95	0.17 ± 0.03	0.758	*t* = 0.322	0.104	*t* = 1.923
Pb	19.74 ± 3.06		21.19 ± 2.91		0.841	*W* = 11		
S	94,917 ± 32445	4,995 ± 233.56	83,238 ± 32,415	4,808 ± 266.14	0.334	*t* = −1.065	0.272	*t* = −1.180
Sb	4.72 ± 0.62		5.99 ± 0.65		0.016**^∗^**	*t* = 3.157		
Si	276.27 ± 75.50	9.25 ± 3.07	287.06 ± 84.44	8.14 ± 0.60	0.594	*t* = 0.555	1	*W* = 12
Sr	97.70 ± 11.66	4.57 ± 0.88	107.94 ± 15.36	5.89 ± 0.76	0.151	*W* = 5	0.035**^∗^**	*t* = −2.540
Ti	5,234 ± 1,833	0.25 ± 0.07	6,564 ± 1,760	0.27 ± 0.05	0.012**^∗^**	*t* = 3.306	0.619	*t* = 3.306
Zn	83.70 ± 48.75	0.03 ± 0.02	98.21 ± 52.66	0.02 ± 0.00	0.233	*t* = 1.302	0.786	*W* = 9

*Mean values and standard deviations are provided (n = 5). Normally distributed data were analyzed using the parametric Student’s oxt-test (t-values). Data not normally distributed were analyzed using the non-parametric Mann–Whitney–Wilcoxon (W-values). Significant differences are indicated (^∗^P < 0.05 and ^∗∗^P < 0.01).*

This set of data indicates that the soil of the tailings dumps is not suitable for revegetation due to its low nutrient content, very low N content (below the detection limit) and slightly alkaline pH. These extreme conditions have been shown to suppress tree root growth and to induce leaf chlorosis and decrease biomass production (Wang et al., 2017). The large amount of Fe and Mn, mostly in oxide forms (Carbonell, unpublished data), may also limit the availability of nutrients at this whole area. Previous studies were carried out on the interactions between plant richness and the physicochemical properties of tailings dumps and have identified pH, metal concentration and bioavailability as the major factors that may limit plant establishment on these sites (Santos et al., 2017).

Disparities between the VS and NVS emerged, as illustrated by the PCA (**Figure 1B**). PCA showed that the set of pedological parameters alone explains approximately 40% of the differences between the two soils. The VS also showed a significantly lower pH and higher CEC (**Table 1**). The differences between the VS and NVS could be due to the presence of the *Betula* trees. The lower pH in the vicinity of the birch roots, which could lead to an increase in the CEC in this area, is probably due to the root metabolic activity. Birch trees are known to exude several acids in the millimolar range, especially as monocarboxylic acids (Sandnes et al., 2005). The birch litter may also slightly contribute to the observed enrichment of the vegetated soil in C and OM.

### Microbial Characteristics

**FIGURE 2:**
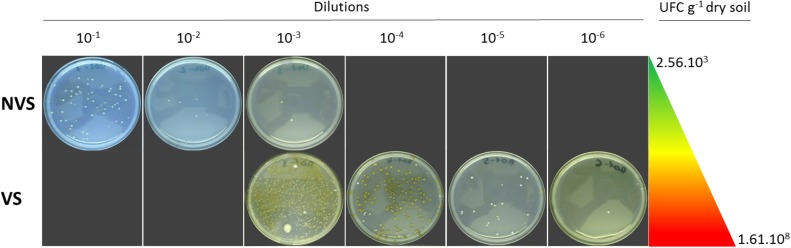
CFU culturable bacterial density expressed as UFC g^−1^ dry soil. The dilution series used to determine the density is represented by the Petri dishes after incubation for the non-vegetated soil (NVS) and the vegetated soil (VS).

The VS fraction had the highest density of culturable bacteria density, which was much higher than in the NVS fraction. In the latter samples, the CFUs were five times lower than the CFUs found in the VS (**Figure 2**). A total of 170 (VS) and 105 (NVS) bacteria were isolated in the present study. Based on their BOX-PCR profiles, the isolates were further placed into 53 (VS) and 43 (NVS) distinct groups and were further identified through comparative sequencing of their 16S rDNA. Isolates were recognized to include a total of 16 different bacterial genera, all of which belonging to four bacterial phyla. The Shannon diversity (H′) index was calculated based on the genera, and similar values were found for the VS (H′ = 1.16) and the NVS (H′ = 1.17).

**FIGURE 3:**
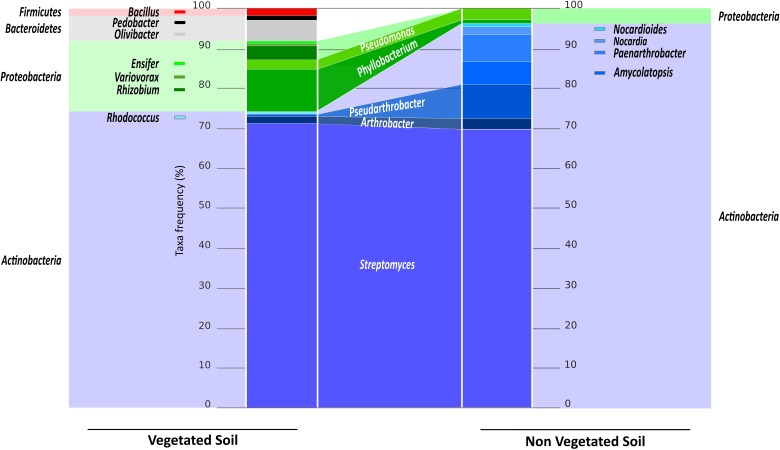
The cultivable bacterial communities from the VS and NVS fractions. The light-colored histograms at the edge represent the abundance of bacterial phyla expressed as a percentage of the total number of bacteria for the VS **(Left)** and the NVS **(Right)**. The dark-colored central histogram represents the abundance of bacterial genera expressed as a percentage of the total number of bacteria for the VS **(Left)** and the NVS **(Right)**. The names in white in the center indicate the shared bacteria between the two habitats.

The phyla *Actinobacteria* and *Proteobacteria* were represented in the two soils, while the *Firmicutes* (including the genus *Bacillus*) and *Bacteroides* (including the genera *Pedobacter* and *Olivibacter*) representatives were found only in the VS fraction. The *Actinobacteria* accounted for more than 95% in the NVS fraction. Within that phylum, the abundance of each genus differed between the two bacterial populations (**Figure 3**). In both soils, *Streptomyces* dominated and accounted for approximately 70% of the total isolates. Additional *Actinobacteria* were found in the NVS (*Amycolatopsis, Nocardia, Nocardioides*, and *Paenarthrobacter*) but were absent from the VS. The *Rhodococcus* isolates were found only in the VS. Within the *Actinobacteria* phylum, the two soils shared only *Pseudoarthrobacter* and *Arthrobacter* (**Figure 3**). The two soils also shared *Proteobacteria* members, although they were represented to a lower degree in the NVS. However, the two soils shared only *Pseudomonas* and *Phyllobacterium* isolates. *Rhizobium, Variovorax*, and *Ensifer* isolates were detected only in the VS fraction.

The *Actinobacteria* phyla dominated the culturable soil community (70% in the VS and 95% in the NVS). These results agree with other data found in the literature. For example, the rhizospheric soil of PTE-hyperaccumulating plant *Thlaspi caerulescens* hosted bacterial communities that were analyzed and compared with that of contaminated bulk soil (Gremion et al., 2003). The sequences belonging to *Actinobacteria* dominated both the bulk and the rhizosphere soils in that study. Isolates from our soils were restricted to the genera *Streptomyces, Arthrobacter*, and *Rhodococcus*, as previously described (Álvarez-López et al., 2015). *Streptomyces* was also isolated from Mn-contaminated soils (Mo et al., 2017) and from the birch rhizosphere collected on the coal mining dump (Ostash et al., 2013). The rhizosphere from the Ni-hyperaccumulating plant *Alyssum serpyllifolium* hosts Ni-resistant bacteria that were predominantly belonging to the *Arthrobacter* and *Streptomyces* genera (Becerra-Castro et al., 2011b). Álvarez et al. (2017) isolated, using culture-dependent methods, and characterized more than 35 *Actinobacteria* genera that were shown to be PTE-tolerant. *Actinobacteria* members tolerant to PTE have been shown to be dominant in PTE-contaminated sites by Margesin et al. (2011), in addition to *Proteobacteria* members, and by Oliveira and Pampulha (2006). Most of these studies agreed on the fact that contaminated soils usually exhibited quantitatively lower culturable bacteria, although *Actinobacteria* were usually less affected by the PTE present in the soil than other culturable heterotrophic bacteria or nitrogen fixers. In addition to *Streptomyces*, *Arthrobacter* was the second most key bacterial genus concerning its PTE-tolerance and thereof it has a real potential for use in bioremediation. Alkaline environments are commonly encountered in soils contaminated with PTE, for instance with Cr. Due to its capacity to tolerate alkaline conditions, Elangovan et al. (2010) suggested the use of both intact *Arthrobacter* cells and cell-free extracts for the bioremediation of alkaline soils contaminated with chromate.

The *Streptomyces* genus, among the order *Actinomycetales* is notably the richest source of natural compounds, including antimetabolites, antibiotics, and antitumor compounds (Bérdy, 2005; Olano et al., 2009; Aigle et al., 2014). For instance, the *Streptomyces* genus produces around 80% of secondary metabolites known to be microbial bioactive compounds (Bérdy, 2005). *Actinobacteria* are able to grow under various life styles such as saprophytes in aquatic environments and soils, or plant commensals such as nitrogen-fixing symbionts. These key features render the *Actinobacteria* well-suited for research-based bioremediation technology.

### Functional Traits of the Bacterial Isolates

**FIGURE 4:**
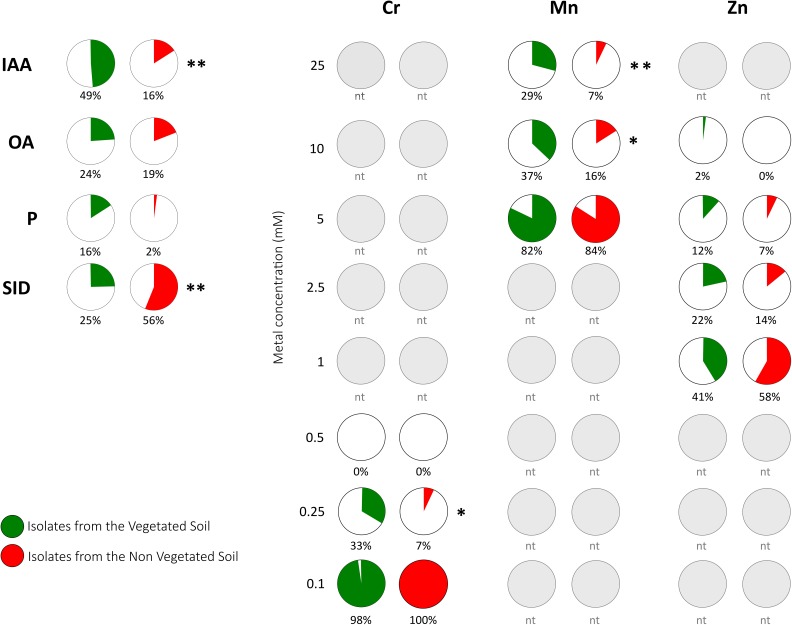
Functional traits of bacterial isolates from the Thann site. Plots representing the percentage of isolates that harbor the positive PGP trait or show metal tolerance. PGP traits were indoleacetic acid (IAA) production, organic acid production (OA), phosphorus solubilization (P), and siderophore production (SID). Metal tolerance was tested for Cr, Mn, and Zn at concentrations ranging from 0.1 to 25 mM. Gray plots indicate non-tested concentrations (nt) for a given metal. Significant differences are indicated (^∗^*P* < 0.05; ^∗∗^*P* < 0.01).

The PGP properties of the bacterial collection comprising 53 (R) and 43 (S) BOX-PCR groups were tested *in vitro* for PGP traits such as IAA production, nutrient uptake, and the metabolism of bacterial compounds regulating plant growth (**Figure 4**). The bacteria isolated from the vegetated soil harbored the PGP bacteria with the higher beneficial potential, with 12% of the isolates exhibiting three or more PGP traits (Supplementary Table S1). The ability to produce IAA was detected in both the isolates from the VS and the NVS fractions, although they were statistically more abundant in the VS. Conversely, the siderophore-producing capacity was higher in the isolates from the NVS. Organic acid-producers and rare phosphate solubilizers were present in both populations and were not significantly different. The production of siderophores by *Streptomyces* isolates has already been shown by Ostash et al. (2013). The genetically and enzymatically based siderophore biosynthesis and transport are well-described in *Streptomyces* (Cruz-Morales et al., 2017). More generally, the *Actinomycetes* group got substantial prominence as PGP microorganism because of its recognized and strong antimicrobial potential and saprophytic behavior dominating numerous soils (Franco-Correa et al., 2010).

In this study, the tolerance to the three metals Zn, Cr, and Mn was investigated (**Table 2**) using the 284 growth medium, and thus our data may only be considered as relative. As indicated in **Figure 4**, the relative order of bacterial toxicity of the three metals was determined as follows: Mn > Zn > Cr. Tolerance to Cr and Zn was considered to be reached at concentrations higher than 0.5 and 1 mM, respectively (Navarro-Noya et al., 2012). In our samples, isolates tolerated Cr concentrations below that threshold that were much lower than that measured for *Cupriavidus metallidurans* (2.5 mM) (Zhao et al., 2012). Conversely, our isolates were more resistant to Zn with an MTC up to 10 mM for some VS isolates. Two percent of our isolates showed an MTC of 10 mM, which is on the same order of magnitude as that determined for the metal-resistant *Cupriavidus metallidurans* (Zhao et al., 2012). Zn-resistant rhizospheric as well as endophytic bacterial isolates of Zn-accumulating *Salix* trees were characterized by Kuffner et al. (2008).

Comparing isolates both from the VS and the NVS samples, the Mn and Cr tolerances were higher for the VS bacteria at the highest concentrations. The strains with the higher metal tolerance in our study were the *Phyllobacterium* sp. WR140 (RO1.15), *Phyllobacterium* sp. WR140 (R01.34) and *Streptomyces* sp. (R04.15), all of which were isolated from the VS fraction. The least tolerant species were isolated from the NVS fraction. The primary tolerant soil bacterium was *Streptomyces flavovirens* (U04.24). To the best of our knowledge, the Mn tolerance in either *Phyllobacterium* or *Streptomyces* has been rarely studied. The *Proteobacteria*, although they were a less abundant species in our isolation experiment, appeared to be the most metal tolerant bacteria (4 over 5 more tolerant). There is abundant literature on Mn oxidation by bacteria (Adams and Ghiorse, 1985; Wang et al., 2009). For instance, the Mn-oxidizing bacterium *Brachybacterium* strain isolated from the deep-sea was able to grow in liquid media supplemented with up to 55 mM MnCl (Wang et al., 2009). Bacterial cells have mechanisms to sense excess metals (Chandrangsu et al., 2017). Generally, the most efficient physiological mechanism that bacteria are exploiting to tolerate excess PTE is efflux. Some of our isolated bacteria (i.e., *Phyllobacterium* sp. WR140) exhibited a Mn MIC higher than that of *Cupriavidus metallidurans* (6 mM) (Zhao et al., 2012). In a previous study, the proportions of metal-tolerant bacterial isolates were primarily represented by Gram-negatives, and the *Proteobacteria* (*Pseudomonas* and *Variovorax* species) dominated (Piotrowska-Seget et al., 2005).

Abbes and Edwerds (1990) evaluated the toxicity of various PTE including Cd, Co, Cu, Cr, Hg, Ni, Zn, and Mn on 34 *Streptomyces* species representative of various taxonomic clusters. Another study described the isolation of several *Streptomyces* strains with resistances to different PTE from contaminated areas, and some exhibited multiple tolerances against different PTE (Álvarez et al., 2013). Due to the extreme abundance of the genus *Streptomyces* in our study, the further characterization of tolerant *Streptomyces* from this red gypsum dump might lead to a better assessment/new discovery of the physiological mechanisms involved in the metal tolerance in this genus and therefore to ecological applications.

However, we examined the tolerance to the Mn ionic form, while the Mn in the Thann soil was primarily in the form of Mn oxides (Zapata, unpublished results). A recent review also pointed out the use of bacteria in Mn biomining processes, describing distinct, taxonomically distant bacteria that have been described to reduce Mn either by enzymatic or non-enzymatic based mechanisms or Das et al. (2011). Mn is usually reduced to fulfill a nutritional need for soluble forms of Mn and Mn-reducing bacteria belong to either aerobes or facultative anaerobes.

### Inoculation Tests

**FIGURE 5:**
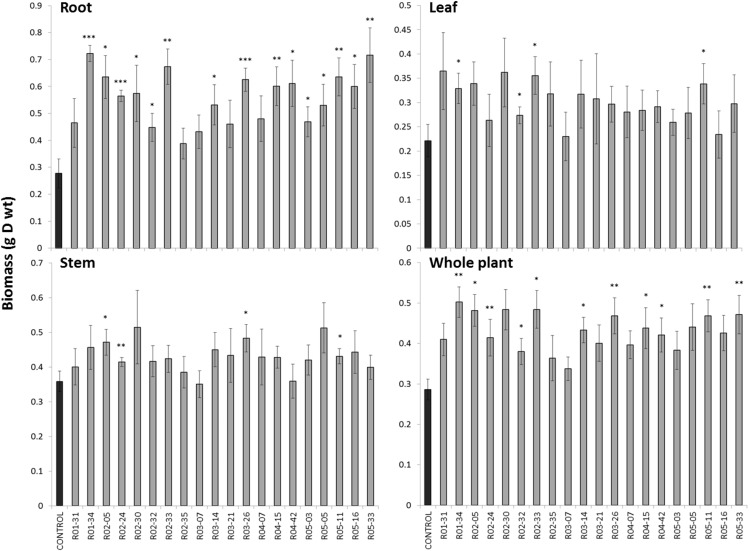
Impact of bacterial inoculation on birch growth. Birch trees were grown for 3 months on the Thann soil inoculated with 20 bacterial isolates collected from the VS fraction. Significant differences are indicated (^∗^*P* < 0.05; ^∗∗^*P* < 0.01; and ^∗∗∗^*P* < 0.001).

A set of 20 bacterial strains isolated from the VS was chosen to carry out an inoculation pot experiment. The bacterial strains were selected from our previous experiments (section “Functional Traits of the Bacterial Isolates”) and those with the best functional traits (PGP traits, siderophore production and metal resistance) were retained, as detailed in Supplementary Table S1. The selected bacterial genera were also representative of the most important phyla in our study and, when possibly, previously tested in the literature. After 3 months of growth, the biomass of the birch plants either inoculated or non-inoculated was analyzed (**Figure 5**). Among the 20 isolates, five increased the total biomass production significantly (*P* < 0.01), and an additional set of six isolates increased the total biomass production significantly (*P* < 0.05) compared with uninoculated control. This was primarily due to an increase in the root biomass, while the shoot biomass was only slightly affected (**Figure 5**). We also measured the elements in birch leaves and found no significant effect of the inoculated bacteria (data not shown).

Among the isolates tested, the *Phyllobacterium* sp. isolate (R01.34) that showed the greatest performance on birch also exhibited multiple PGP traits, including the production of IAA and the solubilization of P. PGP rhizobacteria and bacterial endophytes have huge potential to increase the bioremediation of PTE-contaminated sites (Chen et al., 2010; Luo et al., 2011; Xinxian et al., 2011; Burges et al., 2017). Ma et al. (2013) demonstrated the similar performance of *Phyllobacterium myrsinacearum* on *Sedum plumbizincicola* growth, although this strain increased metal transfer to the shoot, which was not observed in our study. However, another *Phyllobacterium* strain examined in our study did not demonstrate any phytoremediation relevant features, which indicates intragenera variability. A *Pseudomonas* isolate also significantly increased birch biomass production. Similarly, Huang et al. (2016) isolated a Cd-resistant *P. aeruginosa* from a Cd-contaminated oil field, and the inoculations of Cd-polluted soil with that strain significantly elevated the shoot and root biomass. The *Variovorax* isolate (R05-11) also exhibited PGP traits in our study. Similarly, the inoculation of hyperaccumulating plants by *Variovorax paradoxus* isolated from plant rhizosphere significantly increased root biomass (Durand et al., 2018). However, growth promotion was not always linked to functional traits in our study. For instance, *Streptomyces* (R05-33) showed significant growth promotion effects without possessing any significant functional traits. In general, we found that *Streptomyces* strains did not show a great potential to promote plant growth. Other potential traits, such as the release of volatile organic compounds (VOCs), which are very well known in the genus *Streptomyces* (Dias et al., 2017), could be responsible for this beneficial effect. The *Olivibacter soli* (R04-07) isolate exhibited all functional traits while having no growth promoting effects. In greenhouse studies (Alekhya and Gopalakrishnan, 2017), *Cicer arietinum* plant growth was increased by *Streptomyces* due to root length and weight promotion, increase production of nodules, increase production of shoot biomass, pods, and pod weight as compared with the non-inoculated control, demonstrating the colonizing capability of bacteria belonging to this genera.

In addition to the *Arthrobacter* and *Rhodococcus* genera, *Streptomyces* bacteria have received considerable consideration for being used in efficient biotechnological method to clean up contaminated ecosystems. In addition to their physiological diversity, *Streptomyces* isolates may be appropriate for being used as soil inoculants since they are able to rapidly grow as mycelium in semi-selective substrates and their capacity to be genetically transformed (Álvarez et al., 2017). However, most of the studies described in this review paper concerned pesticide-degrading *Actinobacteria*. In the study by Ali et al. (2017), bioremediation was performed on PTE-contaminated mining soils by inoculation using *Streptomyces pactum*. Metal extraction amount data established that the strain Act12 stimulated the PTE uptake and transfer in *Brassica juncea* above ground tissues.

## Conclusion

This study demonstrates that vegetated soils from red gypsum tailings dumps exhibited a higher bacterial diversity compared with non-vegetated soils, based on a culture-dependent method. The number of bacterial isolates having the capacity to produce IAA was higher for bacteria from the vegetated soil, while siderophore production was higher in the bacteria from the non-vegetated soil. Mn and Cr tolerance was also higher for the bacteria isolated from the VS samples. The potential of some bacterial isolates to promote birch growth was observed, although it was not always linked to PGP traits. However, the results of the inoculation tests and the dominance of some *Streptomyces* (within the VS *Actinobacteria* population) and *Phyllobacterium* (within the VS *Proteobacteria* population) associated with *Betula* growing on this soil suggests that those bacteria are involved in the early establishment of woody species in the dump. They appear to be good candidates to identify new approaches for the management of tailings dumps. The *Phyllobacterium* sp. isolate (R01-34) and *Streptomyces* (R05-33) appeared to be promising alternatives for improved inocula and their application at field levels.

## Author Contributions

MC, CZ, VA-L, and NC planned and designed the research. VA-L and CZ performed the experiments and conducted the fieldwork. MC, VA-L, CZ, NC, and CG analyzed the data and wrote the manuscript.

## Conflict of Interest Statement

The authors declare that the research was conducted in the absence of any commercial or financial relationships that could be construed as a potential conflict of interest.

## Footnotes

1 https://www.arb-silva.de/
