# Supplementary material for: Streptomyces Dominate the Soil Under Betula Trees That Have Naturally Colonized a Red Gypsum Landfill
Source: Front Microbiol. 2018 Aug 3;9:1772. doi: 10.3389/fmicb.2018.01772 (PMC6085467; doi:10.3389/fmicb.2018.01772)
Supplement: Supplementary file 1 [file Table_1.DOCX]

**Table S1.** **Detailed functional traits of bacterial isolates.** The bold isolates are those selected for the inoculation experiment (Figure 5).

| Isolate | Code | Group  box | IAA | OA | P | SID | Cr (mM) | | | Mn (mM) | | | Zn (mM) | | | |
| --- | --- | --- | --- | --- | --- | --- | --- | --- | --- | --- | --- | --- | --- | --- | --- | --- |
|  |  |  |  |  |  |  | 0.1 | 0.25 | 0.5 | 5 | 10 | 25 | 1 | 2.5 | 5 | 10 |
| ***Streptomyces sp. 13*** | **R03.14** | **R01** | **-** | **-** | **-** | **+** | **+** | **+** | **-** | **+** | **-** | **-** | **+** | **+** | **+** | **-** |
| ***Streptomyces bobili*** | **R03.07** | **R02** | **+** | **-** | **-** | **+** | **+** | **-** | **-** | **-** | **-** | **-** | **+** | **-** | **-** | **-** |
| *Streptomyces flavofungini* | R03.11 | R03 | - | - | - | - | + | - | - | - | - | - | + | + | - | - |
| ***Streptomyces flavofungini*** | **R05.33** | **R04** | **-** | **-** | **-** | **-** | **+** | **-** | **-** | **-** | **-** | **-** | **+** | **+** | **-** | **-** |
| ***Streptomyces sp. cpRA37*** | **R02.05** | **R05** | **-** | **-** | **-** | **-** | **+** | **-** | **-** | **-** | **-** | **-** | **+** | **-** | **-** | **-** |
| *Streptomyces flavofungini* | R01.18 | R06 | - | - | - | - | + | - | - | - | - | - | + | + | + | - |
| *Streptomyces ederensis* | R05.34 | R07 | - | - | - | - | + | - | - | - | - | - | + | - | - | - |
| *Streptomyces ederensis* | R05.10 | R08 | - | - | - | - | + | - | - | + | - | - | - | - | - | - |
| *Streptomyces ederensis* | R05.08 | R09 | - | - | - | - | + | - | - | + | - | - | + | - | - | - |
| ***Streptomyces phaeochromogenes*** | **R04.42** | **R10** | **-** | **-** | **-** | **-** | **+** | **+** | **-** | **+** | **-** | **-** | **+** | **-** | **-** | **-** |
| ***Streptomyces ederensis*** | **R03.21** | **R11** | **-** | **-** | **-** | **-** | **+** | **-** | **-** | **-** | **-** | **-** | **+** | **+** | **-** | **-** |
| ***Streptomyces sp. SIIB_Zn_R12*** | **R05.16** | **R12** | **+** | **-** | **-** | **-** | **+** | **-** | **-** | **+** | **-** | **-** | **-** | **-** | **-** | **-** |
| *Streptomyces phaeochromogenes* | R05.29 | R13 | - | - | - | - | + | - | - | + | - | - | - | - | - | - |
| *Streptomyces phaeochromogenes* | R02.21 | R14 | - | - | - | - | + | - | - | + | - | - | + | - | - | - |
| ***Streptomyces ederensis*** | **R01.31** | **R15** | **-** | **-** | **-** | **-** | **+** | **-** | **-** | **-** | **-** | **-** | **+** | **-** | **-** | **-** |
| *Streptomyces phaeochromogenes* | R01.19 | R16 | - | - | - | - | + | - | - | - | - | - | + | - | - | - |
| ***Pedobacter sp*** | **R05.03** | **R17** | **+** | **+** | **-** | **-** | **+** | **-** | **-** | **+** | **-** | **-** | **-** | **-** | **-** | **-** |
| *Pseudomonas sp. SJZ* | R01.27 | R18 | + | - | - | - | + | - | - | + | + | - | - | - | - | - |
| *Phyllobacterium myrsinacearum* | R02.04 | R19 | + | + | - | - | + | + | - | + | + | + | - | - | - | - |
| *Olivibacter soli* | R04.13 | R20 | + | + | - | + | + | + | - | + | - | - | + | - | - | - |
| *Olivibacter soli* | R05.14 | R22 | + | + | - | + | + | - | - | + | - | - | - | - | - | - |
| *Phyllobacterium sp. sptzw02* | R05.17 | R23 | + | - | - | - | + | + | - | + | - | - | - | - | - | - |
| ***Phyllobacterium sp. sptzw02*** | **R05.05** | **R24** | **+** | **-** | **-** | **-** | **+** | **+** | **-** | **+** | **-** | **-** | **-** | **-** | **-** | **-** |
| *Phyllobacterium sp. CCBAU 83356* | R04.29 | R25 | + | - | + | - | + | - | - | + | + | + | + | + | - | - |
| ***Olivibacter soli*** | **R04.07** | **R26** | **+** | **+** | **-** | **+** | **+** | **-** | **-** | **+** | **-** | **-** | **-** | **-** | **-** | **-** |
| *Rhizobium radiobacter* | R05.22 | R27 | + | - | + | - | + | + | - | + | + | + | + | + | + | - |
| *Olivibacter soli* | R01.28 | R28 | + | + | - | + | + | - | - | + | - | - | - | - | - | - |
| *Phyllobacterium myrsinacearum* | R05.06 | R29 | + | - | - | - | + | - | - | + | + | + | - | - | - | - |
| *Phyllobacterium myrsinacearum* | R05.02 | R30 | + | - | - | - | + | - | - | + | + | + | - | - | - | - |
| *Pseudarthrobacter sp* | R04.26 | R31 | - | + | - | + | + | + | - | + | + | + | + | - | - | - |
| ***Pseudomonas sp. Q71576*** | **R02.33** | **R32** | **-** | **-** | **+** | **-** | **+** | **-** | **-** | **+** | **+** | **+** | **-** | **-** | **-** | **-** |
| *Phyllobacterium myrsinacearum* | R02.22 | R33 | + | - | + | + | + | + | - | + | + | + | - | - | - | - |
| ***Phyllobacterium sp. WR140*** | **R01.34** | **R35** | **+** | **-** | **+** | **-** | **+** | **-** | **-** | **+** | **+** | **+** | **+** | **+** | **+** | **-** |
| *Phyllobacterium sp. sptzw02* | R05.24 | R36 | + | - | - | - | + | + | - | + | - | - | - | - | - | - |
| *Arthrobacter sp. HBUM179104* | R02.34 | R37 | + | + | - | + | + | + | - | + | - | - | - | - | - | - |
| *Arthrobacter sp* | R02.14 | R38 | - | + | - | + | + | + | - | + | - | - | - | - | - | - |
| *Phyllobacterium sp. WR140* | R01.15 | R39 | - | - | + | + | + | + | - | + | + | + | + | + | + | + |
| *Olivibacter soli* | R05.15 | R40 | + | - | - | - | + | - | - | + | + | - | - | - | - | - |
| ***Variovorax sp. LZA10*** | **R05.11** | **R41** | **+** | **+** | **-** | **-** | **+** | **-** | **-** | **+** | **-** | **-** | **-** | **-** | **-** | **-** |
| ***Sinorhizobium sp. S242*** | **R02.32** | **R42** | **-** | **-** | **-** | **-** | **+** | **-** | **-** | **+** | **+** | **+** | **-** | **-** | **-** | **-** |
| *Pseudomonas reinekei* | R05.13 | R43 | - | - | - | - | + | + | - | + | + | - | - | - | - | - |
| ***Bacillus megaterium*** | **R02.30** | **R44** | **-** | **-** | **-** | **+** | **+** | **+** | **-** | **+** | **+** | **+** | **-** | **-** | **-** | **-** |
| ***Rhodococcus rhodochrous*** | **R02.35** | **R45** | **-** | **-** | **-** | **-** | **+** | **+** | **-** | **+** | **-** | **-** | **+** | **+** | **-** | **-** |
| ***Rhizobium sp. M20*** | **R02.24** | **R46** | **+** | **-** | **-** | **-** | **+** | **-** | **-** | **+** | **-** | **-** | **-** | **-** | **-** | **-** |
| *Pedobacter sp. V48* | R03.15 | R47 | - | + | - | - | + | - | - | + | - | - | - | - | - | - |
| *Phyllobacterium sp. WR140* | R01.24 | R48 | - | - | + | - | + | - | - | + | + | + | - | - | - | - |
| *Phyllobacterium ifriqiyense* | R04.31 | R49 | + | - | + | - | + | - | - | + | + | + | - | - | - | - |
| *Phyllobacterium myrsinacearum* | R03.32 | R50 | - | - | - | - | - | - | - | + | - | - | - | - | - | - |
| ***Arthrobacter sp. Uz1102*** | **R03.26** | **R51** | **-** | **-** | **-** | **+** | **+** | **+** | **-** | **+** | **+** | **-** | **-** | **-** | **-** | **-** |
| *Pseudomonas sp. SJZ* | R01.10 | R52 | + | + | - | - | + | - | - | + | - | - | - | - | - | - |
| ***Streptomyces tricolor*** | **R04.15** | **R53** | **+** | **-** | **-** | **-** | **+** | **-** | **-** | **+** | **+** | **+** | **+** | **+** | **+** | **-** |

| Isolate | Code | Group  box | IAA | OA | P | SID | Cr (mM) | | | Mn (mM) | | | Zn (mM) | | | |
| --- | --- | --- | --- | --- | --- | --- | --- | --- | --- | --- | --- | --- | --- | --- | --- | --- |
|  |  |  |  |  |  |  | 0.1 | 0.25 | 0.5 | 5 | 10 | 25 | 1 | 2.5 | 5 | 10 |
| *Amycolatopsis roodepoortensis* | U04.23 | S01 | - | - | - | + | + | - | - | + | + | + | + | + | - | - |
| *Streptomyces sp. RE2* | U04.15 | S02 | - | - | - | - | + | - | - | - | - | - | + | + | - | - |
| *Streptomyces flavofungini* | U05.06 | S03 | - | - | - | - | + | - | - | + | - | - | + | - | - | - |
| *Streptomyces lomondensis* | U04.13 | S04 | - | - | - | - | + | - | - | - | - | - | + | + | + | - |
| *Streptomyces bobili* | U04.11 | S05 | - | - | - | + | + | - | - | + | - | - | + | - | - | - |
| *Streptomyces sp. AS34* | U01.09 | S06 | + | - | + | + | + | - | - | + | - | - | + | + | + | - |
| *Paenarthrobacter aurescens* | U04.31 | S07 | - | + | - | + | + | - | - | + | - | - | - | - | - | - |
| *Streptomyces bobili* | U03.09 | S08 | - | - | - | - | + | - | - | + | - | - | + | - | - | - |
| *Paenarthrobacter aurescens* | U04.32 | S09 | - | + | - | + | + | - | - | + | - | - | - | - | - | - |
| *Streptomyces sp. RE2* | U01.18 | S10 | - | - | - | - | + | - | - | + | - | - | + | - | - | - |
| *Nocardioides kribbensis* | U04.06 | S11 | - | - | - | + | + | - | - | + | + | - | - | - | - | - |
| *Streptomyces flavovirens* | U04.24 | S12 | - | - | - | + | + | - | - | + | + | - | + | + | + | - |
| *Pseudomonas moraviensis* | U04.16 | S13 | + | - | + | - | + | - | - | + | - | - | - | - | - | - |
| *Paenarthrobacter nitroguajacolicus* | U05.28 | S14 | - | + | - | + | + | - | - | + | - | - | + | - | - | - |
| *Arthrobacter sp* | U04.18 | S15 | - | + | - | + | + | - | - | + | - | - | - | - | - | - |
| *Streptomyces sp. K56(2011)* | U04.01 | S16 | - | - | - | + | + | - | - | + | - | - | - | - | - | - |
| *Streptomyces bobili* | U02.10 | S17 | - | - | - | + | + | - | - | + | - | - | + | - | - | - |
| *Pseudarthrobacter sp* | U02.14 | S18 | + | - | + | + | + | - | - | - | - | - | - | - | - | - |
| *Streptomyces sp. RE2* | U02.09 | S19 | - | - | - | - | + | - | - | + | - | - | + | - | - | - |
| *Pseudarthrobacter sp* | U05.25 | S20 | + | - | + | + | + | - | - | + | - | - | - | - | - | - |
| *Streptomyces bobili* | U01.16 | S21 | - | - | - | - | + | - | - | + | - | - | + | - | - | - |
| *Arthrobacter sp. Tibet-YD4524-4* | U01.12 | S22 | + | + | + | + | + | + | - | - | - | - | - | - | - | - |
| *Streptomyces flavoviridis* | U05.17 | S23 | - | - | - | - | + | - | - | - | - | - | + | - | - | - |
| *Streptomyces bobili* | U05.08 | S24 | - | + | - | + | + | - | - | - | - | - | - | - | - | - |
| *Streptomyces sp. Ds10* | U05.18 | S25 | - | - | - | - | + | - | - | + | - | - | + | - | - | - |
| *Paenarthrobacter aurescens* | U04.10 | S26 | - | + | - | + | + | + | - | + | - | - | - | - | - | - |
| *Pseudarthrobacter sp* | U03.11 | S27 | - | - | - | + | + | - | - | + | - | - | - | - | - | - |
| *Streptomyces phaeochromogenes* | U05.22 | S28 | - | - | - | - | + | - | - | + | - | - | + | - | - | - |
| *Streptomyces bobili* | U03.07 | S29 | + | - | + | - | + | - | - | + | - | - | + | - | - | - |
| *Streptomyces bobili* | U02.16 | S30 | - | - | - | + | + | - | - | + | + | - | + | - | - | - |
| *Streptomyces sp. VTT E-052903* | U05.15 | S31 | - | - | - | - | + | - | - | + | - | - | + | - | - | - |
| *Nocardia sp.* | U02.06 | S32 | - | - | - | - | + | - | - | + | - | - | - | - | - | - |
| *Phyllobacterium sp. CCBAU 83356* | U05.34 | S33 | + | - | + | - | + | - | - | + | - | - | - | - | - | - |
| *Amycolatopsis coloradensis* | U05.11 | S34 | - | - | - | + | + | - | - | + | + | + | + | - | - | - |
| *Amycolatopsis sp. K6-08* | U05.24 | S35 | - | - | - | + | + | - | - | + | + | + | + | + | - | - |
| *Pseudarthrobacter sp* | U05.12 | S36 | - | - | - | + | + | - | - | - | - | - | - | - | - | - |
| *Streptomyces sp. RE2* | U02.17 | S37 | - | - | - | - | + | - | - | + | - | - | + | - | - | - |
| *Streptomyces bobili* | U05.09 | S38 | - | - | - | + | + | - | - | + | - | - | + | - | - | - |
| *Streptomyces bobili* | U02.05 | S39 | - | - | - | + | + | - | - | + | - | - | + | - | - | - |
| *Streptomyces bobili* | U01.03 | S40 | - | - | - | - | + | - | - | + | - | - | + | - | - | - |
| *Paenarthrobacter aurescens* | U04.17 | S41 | - | + | - | + | + | + | - | + | + | - | - | - | - | - |
| *Streptomyces lomondensis* | U05.33 | S42 | - | - | - | - | + | - | - | + | - | - | - | - | - | - |
| *Streptomyces sp. 19504* | U01.02 | S43 | - | - | - | - | + | - | - | + | - | - | - | - | - | - |
